# Parity and Cardiovascular Disease Mortality: a Dose-Response Meta-Analysis of Cohort Studies

**DOI:** 10.1038/srep13411

**Published:** 2015-08-24

**Authors:** Haichen Lv, Hongyi Wu, Jiasheng Yin, Juying Qian, Junbo Ge

**Affiliations:** 1 Department of Cardiology, First Affiliated Hospital of Dalian Medical University, 222 Zhongshan Road, Dalian, Liaoning, China, 116011; 2 Shanghai Institute of Cardiovascular Diseases, Department of Cardiology, Zhongshan Hospital, Fudan University, 180 Fenglin Road, Shanghai, China, 200032

## Abstract

Parity has been shown to inversely associate with cardiovascular disease (CVD) mortality, but the evidence of epidemiological studies is still controversial. Therefore, we quantitatively assessed the relationship between parity and CVD mortality by summarizing the evidence from prospective studies. We searched MEDLINE (PubMed), EMBASE and ISI Web of Science databases for relevant prospective studies of parity and CVD mortality through the end of March 2015. Fixed- or random-effects models were used to estimate summary relative risks (RRs) and 95% confidence intervals (CIs). Heterogeneity among studies was assessed using the *I*^2^ statistics. All statistical tests were two-sided. Ten prospective studies were included with a total of 994,810 participants and 16,601 CVD events. A borderline significant inverse association was observed while comparing parity with nulliparous, with summarized RR = 0.79 (95% CI: 0.60–1.06; *I*^2^ = 90.9%, *P* < 0.001). In dose-response analysis, we observed a significant nonlinear association between parity number and CVD mortality. The greatest risk reduction appeared when the parity number reached four. The findings of this meta-analysis suggests that ever parity is inversely related to CVD mortality. Furthermore, there is a statistically significant nonlinear inverse association between parity number and CVD mortality.

Cardiovascular disease (CVD) is one of the dominant causes of death and disability. It accounts for millions deaths worldwide each year and leads to be an important public issue^1^. Apart from age, hypertension, hyperlipidemia, diabetes, obesity, and smoking, which are the most established risk factors for CVD, recent studies have hypothesized that sex hormone may play a role in the etiology of CVD^1^,^2^. Several studies have demonstrated that males are two to three times more likely to die prematurely from cardiovascular disease than females. This ratio persists in all countries independent of their overall death rate from this condition^2^. This theory may be affected by complex influences from sex hormone.

Pregnancy and parturition are important events in the life of a woman. Fluctuations of serum sex hormone levels during the process of pregnancy and delivery, perinatal hemodynamic changes, oxidative stress and other gestational factors exert complex influences on the cardiovascular system. Although previous studies have devoted great efforts to evaluate the relationship between childbearing history and mortality of CVD, the aforementioned association is still inconsistent. The association between parity and risk of CVD was first studied in the 1980s, which demonstrated the increased prevalence of CVD with parity number^3^. However, subsequent studies found minimal or no evidence for the aforementioned association^3^,^4^,^5^,^6^,^7^,^8^,^9^. Given the inconsistency of previous findings, we therefore carried out this systematic review and meta-analysis to summarize the evidence of the relationship between parity and CVD mortality.

## Results

### Study characteristics and quality assessment

Table 1 demonstrated the characteristics of the ten included prospective studies^10^,^11^,^12^,^13^,^14^,^15^,^16^,^17^,^18^,^19^, in which 16,601 cases and 978,209 non-cases was represented. Among the ten included studies, five were carried out in the United States^11^,^15^,^17^,^18^,^19^, and one for each in Israel^10^, Australia^12^, China^14^, Korea^13^, and Finland^16^. Cohort sizes ranged from 867^18^ to 585,455^17^, and the number of CVD cases varied from 45^18^ to 7125^14^.

The information of study quality is described in Supplementary Table S1. Briefly, three studies^13^,^14^,^15^ were not assigned a star in the column of “representativeness of the exposed cohort” because these studies utilized special populations. Three studies^13^,^16^,^17^ were assigned two stars in the column of “control for important factor or additional factor” because they adjusted for more than two important confounders in the primary analyses. Two studies^17^,^18^ were not assigned a star in the column of “follow-up long enough for outcomes to occur” because the follow-up periods were less than ten years. Three studies^12^,^13^,^19^ were not assigned a star in the column of “adequacy of follow-up of cohorts” because the follow-up rates were less than 75%.

### Ever parity *versus* nulliparous

**Figure 1:**
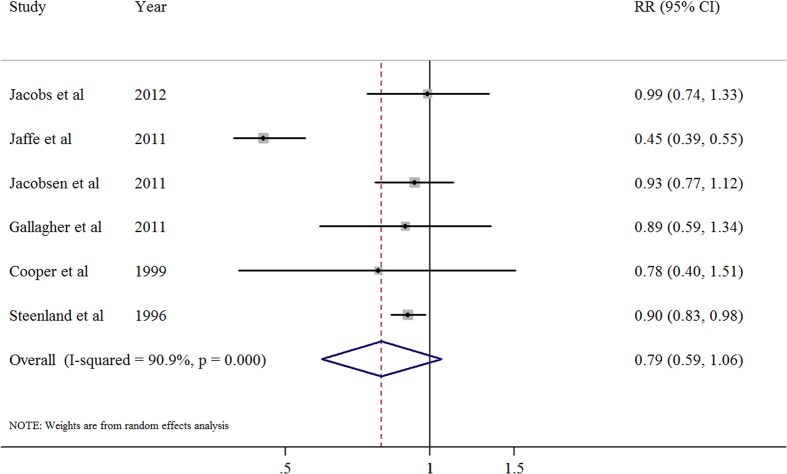
Association between ever parity and cardiovascular disease mortality.

Six studies^11^,^14^,^15^,^17^,^18^,^19^ focused on the relationship between ever parity and CVD mortality. The summary relative risk of CVD for the ever parity compared with nulliparous was 0.79 (95% CI, 0.60–1.06), with significant heterogeneity (*I*^2^ = 90.9%; *P* < 0.001; Fig. 1). We observed no publication bias through Egger’s test (*P* = 0.760) or Begg’s test (*P* = 0.260) (Supplementary Figure S1).

### Dose-response analysis of parity number

**Figure 2:**
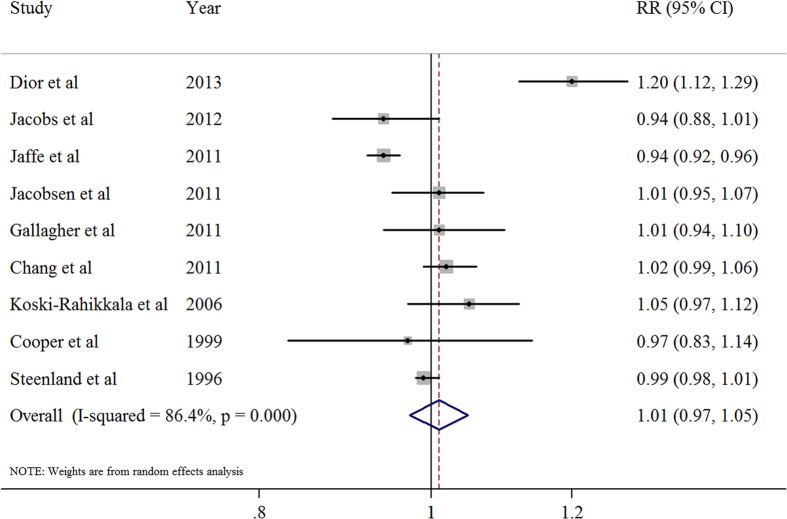
The relative risk(RR) for per live birth.

**Figure 3:**
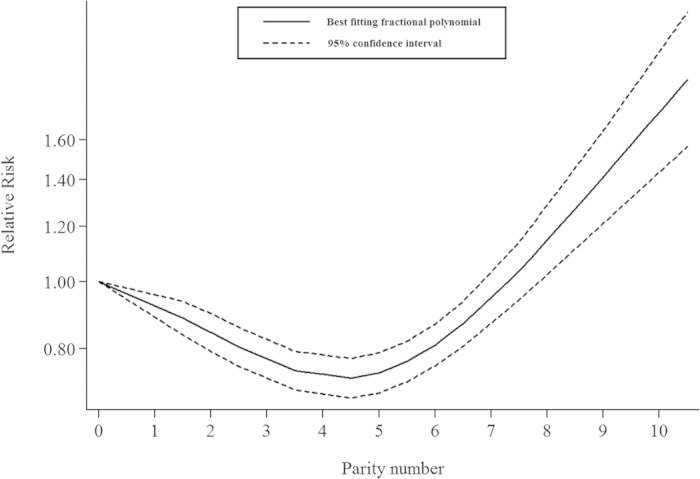
Dose-response analysis of parity number.

We included nine prospective studies^10^,^11^,^13^,^14^,^15^,^16^,^17^,^18^,^19^ in the dose-response analysis. The summary risk estimates for per live birth was 1.01 (95% CI, 0.97–1.05), with significant heterogeneity (*I*^2^ = 86.4%; *P* < 0.001; Fig. 2). In addition, we observed a significant nonlinear relationship between parity number and CVD mortality (*P* < 0.001). There was evidence of a J-shaped association in the non-linear dose-response meta-analysis of parity number and CVD mortality. The association of parity number with CVD mortality appears to follow an inversely linear dose-response pattern until the parity number reaches four live births. After this point, the association appears to rebound (Fig. 3).

### Subgroup and sensitivity analysis

In the subgroup analysis of per one live birth and CVD mortality, we found non-significant results in the majority of the strata. However, there was a significant difference among the summarized results of the studies whether adjustment for cigarette smoking (Table 2), which might be partially responsible for the significant heterogeneity of the main result.

In the sensitivity analysis, the six study-specific RR of the ever parity *versus* nulliparous ranged from a low of 0.76 (95% CI, 0.54–1.06; *I*^2^ = 92.5%; *P* < 0.001) after omission of the study by Jacobs and colleagues^11^ to a high of 0.91 (95% CI, 0.85–0.98; *I*^2^ = 0%; *P* = 0.957) after omission of the study by Jaffe and colleagues^19^. Additionally, The 9 study-specific RR of the parity number ranged from a low of 0.99 (95% CI, 0.96–1.02; *I*^2^ = 77.1%; *P* < 0.001) after omission of the study by Dior and colleagues^10^ to a high of 1.02 (95% CI, 0.98–1.06; *I*^2^ = 80.0%; *P* < 0.001) after omission of the study by Jaffe and colleagues^19^,^20^.

## Discussion

Findings from this meta-analysis demonstrate a borderline significant reduced risk of CVD mortality in ever parity *versus* nulliparous women (RR = 0.79, 95% CI: 0.60–1.06). Additionally, non-linear dose-response analysis displays a potential J-shaped relationship between parity number and CVD mortality. We firstly comprehensively and quantitatively evaluate the relationship between parity and CVD mortality.

Several potential mechanisms might have been proposed but the exact biologic mechanisms are not fully understood. Previous studies showed that complicated metabolic changes, such as dyslipidemia, abnormal glucose tolerance and increased body mass index, developed with increasing parity number^20^,^21^. Besides, as pregnancy itself can be regarded as a state of increased insulin resistance, recurrent pregnancies may result in an additive effect on the later insulin resistance, which may give an explanation to the positive association between high (>4) parity and cardiovascular mortality^16^. The protective effect from moderate parity for CVD may link with the enhanced endothelial function in pregnancy, which results in greater bioavailable nitric oxide^11^. It is worth mentioning that increased endothelial function from pregnancy, unlike its concurrent metabolic change and other temporary disorders, may continue postpartum^22^.

As for the associations between endogenous estrogen exposure and CVD mortality, however, former studies have reported conflicting results^13^. Numerous pregnancies always result in prolonged exposure to high levels of estrogen and progesterone, which may reduce the risk of CVD. However, numerous pregnancies also relate to older maternal age, inflammation and oxidative stress, which are tightly associated with adverse predictors to CVD. It is also possible that higher fertility may reflect women who are better in general and therefore at relatively lower risk for CVD mortality^11^. However, increased risk of CVD was more likely to be attributable to lifestyle factors such as anxiety, stress, even fear of raising children^6^,^13^. Socioeconomic factors may also enhance the relationship between parity and CVD mortality, because both CVD and high parity have higher frequency in lower social classes^10^.

Compared to nulliparous, our meta-analysis shows that ever parity has a significantly protective effect against the death caused by CVD within a limited number of parity. Interestingly, as the 10 included cohorts displayed different viewpoints of parity and CVD mortality, the published information on this issue is scarce and inconsistent. Some studies reported a non-linear association between parity and CVD mortality^10^,^19^. For example, Dior *et al.*^10^ reported that higher mortality rates were observed for mothers of 1 child (HR = 1.18; 95% CI, 1.04–1.4), mothers of 5–9 children (HR = 1.21; 95% CI, 1.09–1.33), and mothers of ≥10 children (HR = 1.49; 95% CI,1.12–1.99) than mothers of 2–4, which was similar to the findings of present study. Additionally, Gallagher *et al.*^14^ showed slightly increased risk of CVD mortality associated with more than five births while Jaffe confirmed that the risk estimates of CVD mortality were higher among women with no children (HR 2.43, CI 1.49, 3.96) and women with more than 8 children (HR 1.64, CI 1.02, 2.65) than those with two children^19^,^20^. In contrast, a cohort study reported by Jacobs *et al.* suggested that women with more than 4 pregnancies were at lower CVD mortality risk^11^, with further reduction of mortality as parity increases^12^. Conversely, several studies denied the direct association between parity and CVD mortality^13^,^15^. These conflicting results from previous investigations make the present study more meaningful. Possible reasons for this discrepancy include a variation of cohort size, different races, subgroup analysis and so on. The results of the present study suggest that women with 4 or 5 children have the lowest risk of CVD induced mortality.

When we carried out the analysis of parity and CVD mortality by geographic location, significant positive association were observed in Israelite^10^. Considering limited studies from other European countries(Table 1), the interpretation of the results should be taken restrainedly. Although more American studies focused on this topic^11^,^15^,^17^,^18^,^19^, the results were still confusing. Nevertheless, we found few studies from Asia and only two of them focused on this topic from China^14^ and Korea^13^, which both showed borderline relationship between parity and CVD mortality without statistical significance. However, comparing with the reports from northern hemisphere, Simons and colleagues^12^, from Australia, reported that increased parity was associated with a tendency for decreased risk of CVD mortality, but without notably statistical significance either.

Our meta-analysis has several strengths. The included studies showed conflicting results, which may result from their limited statistical range and power. Our study, however, was conducted based on approximately 994,810 study participants and 16,601 CVD cases from 10 cohort studies. This massive database generated a stronger statistical power for us to detect and verify this putative association. Moreover, prospective design also helped to make the present study more robust by eliminating selection bias and recall bias. At the same time, our approach with meta-analysis toward the studies was one of the powerful tools to assess the role of parity in the risk of CVD mortality. To guarantee the analysis quality, this meta-analysis had a big sample size, and the follow-up duration was considerably long. Although outcome evidence from long-term randomized trials is ideal, these studies are too difficult to implement on a practical basis, especially regarding reproductive factors.

Meanwhile, several limitations should also be acknowledged. First, the discovered information toward the relationship between parity and mortality from subtypes of CVD and each diagnosis criteria was limited, which remained this topic open to further research in the field of observational cohort studies. Second, parity is associated with several other factors, such as gestational hypertension, gestational diabetes mellitus, gynecological tumor and so on, which are established risk factors for CVD. Third, higher deliveries was generally associated with unhealthy factors such as a higher possibility of abortion, oral contraceptive and so on. The observed association between moderate parity and a lower risk of CVD is unlikely to consider these confounders. Fourth, delivery mode was not taken into account in our study., natural labor and caesarean section might lead to different prognoses. Last, the quality of individual original studies varied. Quality scoring might ignore some important information or introduce somewhat arbitrary subjective factors into the analysis because of combining disparate study features into a single score. Although we used the Newcastle-Ottawa Scale (NOS)^23^ to assess the quality of included studies, we did not score these studies or describe them as high or low quality quantitatively. Studies included in this review were all prospective studies and met most validity criteria, but three of them^13^,^14^,^15^ utilized special populations, four studies^14^,^15^,^18^,^19^ adjusted for limited confounders in the primary analyses, two studies^17^,^18^ didn’t follow up for at least ten years, and the follow-up rates of three studies^12^,^13^,^19^ were less than 75%. These flaws might bring some bias, which should be paid more attention by investigators in the future. We have observed a significant heterogeneity across studies in our analysis pool, showing notable clinical relevance. We believe this may result from our large sample size, which can confer greater statistical power to heterogeneity tests.

In conclusion, the present meta-analysis suggests a potential non-linear J-shaped relationship between the number of parity and CVD mortality. Since the number of included studies was limited, further studies are warranted to confirm our findings as well as to stratify the results by the types of CVD and other risk factors to eliminate the residual confounding.

## Methods

### Search Strategy

We applied the Meta-analysis Of Observational Studies in Epidemiology (MOOSE) as the guidance to conduct and report this study^24^. A holistic review of the published articles (through the end of March 2015) with limitation to humans was performed by using MEDLINE (PubMed), EMBASE and ISI Web of Science databases. We used the following search strategy and keywords: (reproduction or reproductive factors or livebirth or pregnancy or parity) and (cardiovascular diseases or coronary heart disease) and (cohort study or prospective study). Furthermore, the listed article references were also examined and analyzed for additional studies.

### Study Selection

**Figure 4:**
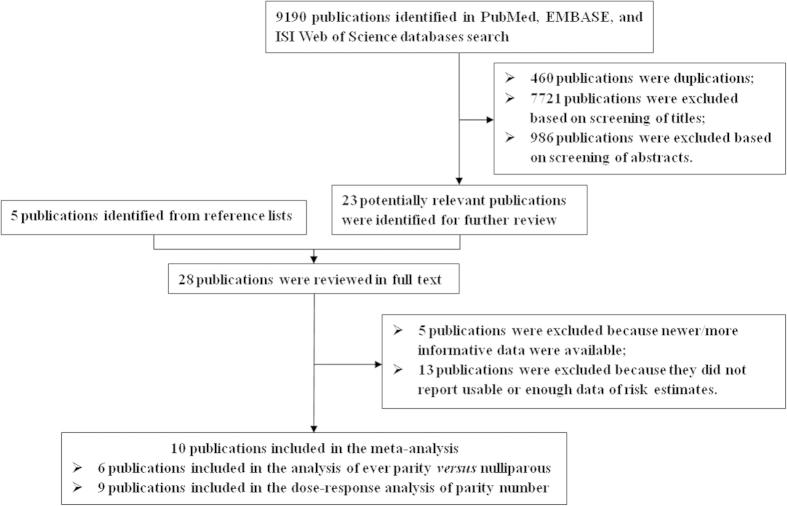
Selection of studies for inclusion in meta-analysis.

The literature was selected by 4 criteria: (1) the study had a prospective design; (2) the exposure was either parity or the number of livebirth; (3) the outcome was mortality from CVD, coronary heart diseases (CHD), or ischemic heart disease (IHD); and (4) risk estimates with 95% confidence intervals (CIs). If multiple literatures were based on the same population, we chose the literature with larger sample size. We identified 10 potentially relevant prospective studies^10^,^11^,^12^,^13^,^14^,^15^,^16^,^17^,^18^,^19^ from 7973 articles (Fig. 4). Four studies^10^,^12^,^13^,^16^ only reported the results of highest compared with lowest number of livebirth, which were only brought into the analysis of parity number.

### Data Extraction

Two independent investigators (Haichen Lv and Hongyi Wu) evaluated the eligibility and abstracted the data of each study. Discrepancies were settled by discussion. We summarized the following data from the selected studies: name of first author, year of publication, study’s country and design, sample size, follow-up year, exposure and outcome method, adjusted risk estimates and their 95%CIs of each included study for ever parity compared with nulliparous, and potential confounders adjusted for in the primary analysis. We also calculated the risk estimate from the raw data demonstrated in the literature^19^ when it was not presented in the study.

### Quality Assessment

The Newcastle-Ottawa Scale (NOS)^23^ included 3 quality parameters for cohort studies: the selection of study groups, comparability of groups and ascertainment of either the exposure or outcome of interest was used by two independent researchers (Haichen Lv and Hongyi Wu) to assess study quality.

### Statistical Analysis

Considering heterogeneity between the studies, we used fixed-effects^25^ or the random-effects model^26^ to calculate summary risk estimate and 95% CIs for the ever parity compared with nulliparous and for the dose-response analysis. The method proposed by Hamling *et al.*^27^ was used to recalculate RRs for studies^10^,^11^,^14^,^15^,^16^,^17^ that did not use the category with the lowest number of parity as the reference or those that reported the risk estimates of each exposed category instead of combined estimates.

The methods described by Greenland *et al.*^28^ and Orsini *et al.*^29^ were used for the dose-response analysis, which require the distribution of cases and person-years or non-cases and the RRs with the variance estimates for at least three quantitative exposure categories^30^. We assigned the median or mean level of parity number in each category to the corresponding RR for each study demonstrated in the literature. We estimated the mean duration in each category by calculating the average of the lower and upper bound for studies that reported parity number by ranges of duration. We made the assumption that the open-ended interval length was the same as the adjacent interval when the highest category was open-ended. We set the lower bound to zero. We examined a potential nonlinear dose-response relationship between parity number and CVD risk using fractional polynomial models^31^, when the lowest category did not have a lower bound. We determined the best-fitting second-order fractional polynomial regression model, defined as the one with the lowest deviance. We used a likelihood ratio test to assess the difference between the nonlinear and linear models to test for nonlinearity^32^. The dose-response results are presented at one live birth increments. Finally, we carried out a sensitivity analysis by excluding each study in turn to evaluate the impact of individual data set on the overall estimate.

In the studies, heterogeneity was assessed by using the *I*^2^ statistics^25^. Subgroup analyses were carried out based on the CVD case number (≤500 versus >500), geographic location (North America, Europe, and Asia), and whether adjustment for potential confounders. Small study bias, such as publication bias, was evaluated via Egger’s test^33^, Begg’s test^34^, and funnel plots. All statistical analyses were conducted via Stata software (version 11.2; StataCorp). *P* values were two sided with a significance level of 0.05.

## Additional Information

**How to cite this article**: Lv, H. *et al.* Parity and Cardiovascular Disease Mortality: a Dose-Response Meta-Analysis of Cohort Studies. *Sci. Rep.* **5**, 13411; doi: 10.1038/srep13411 (2015).

## Supplementary Material

Supplementary Information

## Figures and Tables

**Table 1:** Characteristics of studies of parity and CVD mortality.

Authors	Year	Location	Studydesign	Gender	No. ofcases	No. ofsubject	Age (years)	Duration offollow-up(years)	Meanage atfirstbirth(year)	Exposure categories(exposure/caseassessment)	HR/RR (95% CI)	Matched/Adjustedfactors
Dior *et al.*^10^†	2013	Israel	CS	Female	386	40,454	23.8–60.9y	37y	23.8y	CHD: ≥10 vs. 1 (Questionnaire/Population registry)	5.40 (1.97–14.85)	Age at first birth, mother’s origin, socioeconomic status, diabetes mellitus, gestational diabetes mellitus, toxemia, hypertension, smoking, Cesarean sections
Simons *et al.*^12^	2012	Australia	CS	Female	N/A	1571	Mean, 69.6y	16y	N/A	CHD: ≥6 vs. Nulliparous (Questionnaire/Death records)	1.34 (0.68–2.66)	Alcohol intake, smoking, peak expiratory flow, physical disability, self-rated health and atrial fibrillation
Jacobs *et al.*^11^†	2012	United States	CS	Female	523	1294	50–69y	19.3y	N/A	CVD: Ever parous vs. Nulliparous ≥4 vs. Nulliparous CHD: Ever parous vs. Nulliparous ≥4 vs. Nulliparous (Questionnaire/Death registry)	0.99 (0.74–1.33) 0.63 (0.40–0.99) 1.29 (0.84–1.98) 0.87 (0.45–1.71)	Age, years postmenopause, BMI, and high-density lipoprotein cholesterol
Jaffe *et al.*^19^§	2011	United States	CS	Female	1857	62,822	45–89y	9y	N/A	CVD: Ever parous vs. Nulliparous ≥8 vs. Nulliparous (Questionnaire/Death registry)	0.45 (0.39–0.55) 0.77 (0.59–0.98)	N/A
Jacobsen *et al.*^15^†	2011	United States	CS	Female	1149	19,688	≥25y	10.7y	N/A	IHD: Ever parous vs. Nulliparous ≥5 vs. Nulliparous (Questionnaire/Death registry)	0.93 (0.77–1.12) 1.12 (0.78–1.59)	Marital status, level of education, and age at first delivery (parity number)
Gallagher *et al.*^14^†	2011	China	CS	Female	7125	267,400	≥30y	11y	N/A	IHD: Ever parous vs. Nulliparous ≥5 vs. Nulliparous (Questionnaire/Death registry)	0.89 (0.59–1.34) 1.05 (0.55–2.01)	Age
Chang *et al.*^13^	2011	Korea	CS	Female	478	3257	Mean, 66.8y	20y	21.3y	CVD: ≥8 vs. 0–4 CHD: ≥8 vs. 0–4 (Questionnaire/Death records)	1.20 (0.94–1.54) 1.80 (0.82–3.98)	Age at entry, BMI, hypertension, drinking, smoking, education, and occupation
Koski-Rahikkala *et al.*^16^†	2006	Finland	CS	Female	251	12002	49–83y	35y	22.8y	CVD: Parity: ≥10 vs. 1 (Questionnaire/Death records)	2.33 (0.69–7.87)	Age, socioeconomic position, pre-pregnancy BMI, smoking before pregnancy, age at menarche and age at first birth
Cooper *et al.*^18^	1999	United States	CS	Female	45	867	63–81y	5y	N/A	IHD: Ever parous vs. Nulliparous Parity: ≥4 vs. Nulliparous (Questionnaire/Death records)	0.78 (0.40–1.51) 0.88 (0.40–1.97)	Age
Steenland *et al.*^17^†	1996	United States	CS	Female	4787	585,455	≥30y	8y	N/A	CHD: Ever parous vs. Nulliparous Parity: ≥6 vs. Nulliparous (Questionnaire/Death records)	0.90 (0.83–0.98) 0.94 (0.83–1.08)	Age, race, smoking, baseline health status, blue collar status, education, exercise, hypertension medication use, BMI, estrogen use, and vegetable consumption.

BMI: body mass index; CHD: coronary heart disease; CI: confidence interval; CS: cohort study; CVD: cardiovascular disease; IHD: ischemic heart disease; N/A: not available; RR: relative risk.

^†^Recalculate the RR by the method proposed by Hamling *et al.*

^§^Odds ratio and 95% CI calculated from published data using EpiCalc 2000.

**Table 2:** Summary risk estimates of the association between parity number and cardiovascular disease mortality (per 1 live birth).

	**No. of**	**Summary RR**	***I*^2^ Value**	*P*_h_*
	**studies**	**(95% CIs)**	**(%)**	
Overall	9	1.01 (0.97–1.05)	86.4	<0.001
Subgroup analyses				
Number of cases				
≤500	4	1.07 (0.98–1.16)	82.8	0.001
>500	5	0.97 (0.94–1.01)	78.3	0.001
Geographic Location				
North America	5	0.97 (0.94–1.01)	77.3	0.001
Europe	1	1.05 (0.98–1.12)	N/A	N/A
Asia	3	1.07 (0.97–1.19)	88.5	<0.001
Adjustment for potential confounders				
Age				
Yes	7	1.03 (0.98–1.07)	82.2	<0.001
No	2	0.97 (0.90–1.04)	79.9	0.026
BMI				
Yes	4	1.00 (0.97–1.03)	58.5	0.065
No	5	1.02 (0.93–1.12)	91.3	<0.001
DM				
Yes	1	1.20 (1.12–1.29)	N/A	N/A
No	8	0.99 (0.96–1.02)	75.2	<0.001
Hypertension				
Yes	3	1.06 (0.98–1.14)	93.0	<0.001
No	6	0.98 (0.94–1.03)	65.2	0.013
Alcohol drinking				
Yes	1	1.02 (0.99–1.06)	N/A	N/A
No	8	1.01 (0.97–1.05)	87.2	<0.001
Cigarette smoking				
Yes	4	1.05 (0.99–1.12)	90.0	<0.001
No	5	0.95 (0.93–0.97)	46.7	0.112

BMI: body mass index; CI: confidence interval; DM: diabetes mellitus; N/A: not available; RR: relative risk.

^*^P value for heterogeneity within each subgroup.

## References

[b1] Bonow R. O., Smaha L. A., Smith S. J., Mensah G. A. & Lenfant C. World Heart Day 2002: The International Burden of Cardiovascular Disease: Responding to the Emerging Global Epidemic. Circulation. 106, 1602–1605 (2002). 12270848 10.1161/01.cir.0000035036.22612.2b

[b2] Jones T. H. Testosterone Deficiency: A Risk Factor for Cardiovascular Disease? Trends Endocrinol Metab. 21, 496–503 (2010). 20381374 10.1016/j.tem.2010.03.002

[b3] Colditz G. A. *et al.* A Prospective Study of Age at Menarche, Parity, Age at First Birth, and Coronary Heart Disease in Women. Am J Epidemiol. 126, 861–870 (1987). 3661534 10.1093/oxfordjournals.aje.a114723

[b4] Barrett-Connor E. *et al.* Factors Associated with Glucose and Insulin Levels in Healthy Postmenopausal Women. Diabetes Care. 19, 333–340 (1996). 8729156 10.2337/diacare.19.4.333

[b5] Skilton M. R., Serusclat A., Begg L. M., Moulin P. & Bonnet F. Parity and Carotid Atherosclerosis in Men and Women: Insights Into the Roles of Childbearing and Child-Rearing. Stroke. 40, 1152–1157 (2009). 19211493 10.1161/STROKEAHA.108.535807

[b6] Lawlor D. A. *et al.* Is the Association Between Parity and Coronary Heart Disease Due to Biological Effects of Pregnancy Or Adverse Lifestyle Risk Factors Associated with Child-Rearing? Findings From the British Women’s Heart and Health Study and the British Regional Heart Study. Circulation. 107, 1260–1264 (2003). 12628945 10.1161/01.cir.0000053441.43495.1a

[b7] Atsma F. *et al.* Reproductive Factors, Metabolic Factors, and Coronary Artery Calcification in Older Women. Menopause. 15, 899–904 (2008). 18779678 10.1097/gme.0b013e3181653d7d

[b8] Catov J. M. *et al.* Parity and Cardiovascular Disease Risk Among Older Women: How Do Pregnancy Complications Mediate the Association? Ann Epidemiol. 18, 873–879 (2008). 19041585 10.1016/j.annepidem.2008.09.009 PMC2614660

[b9] Bertuccio P., Tavani A., Gallus S., Negri E. & La Vecchia C. Menstrual and Reproductive Factors and Risk of Non-Fatal Acute Myocardial Infarction in Italy. Eur J Obstet Gynecol Reprod Biol. 134, 67–72 (2007). 17303313 10.1016/j.ejogrb.2007.01.005

[b10] Dior U. P. *et al.* Association Between Number of Children and Mortality of Mothers: Results of a 37-Year Follow-Up Study. Ann Epidemiol. 23, 13–18 (2013). 23176782 10.1016/j.annepidem.2012.10.005 PMC3520435

[b11] Jacobs M. B., Kritz-Silverstein D., Wingard D. L. & Barrett-Connor E. The Association of Reproductive History with All-Cause and Cardiovascular Mortality in Older Women: The Rancho Bernardo Study. Fertil Steril. 97, 118–124 (2012). 22130321 10.1016/j.fertnstert.2011.10.028 PMC3245788

[b12] Simons L. A., Simons J., Friedlander Y. & McCallum J. Childbearing History and Late-Life Mortality: The Dubbo Study of Australian Elderly. Age Ageing. 41, 523–528 (2012). 22459707 10.1093/ageing/afs016

[b13] Chang H. S., Odongua N., Ohrr H., Sull J. W. & Nam C. M. Reproductive Risk Factors for Cardiovascular Disease Mortality Among Postmenopausal Women in Korea: The Kangwha Cohort Study, 1985–2005. Menopause. 18, 1205–1212 (2011). 21659906 10.1097/gme.0b013e31821adb43

[b14] Gallagher L. G. *et al.* Reproductive History and Mortality From Cardiovascular Disease Among Women Textile Workers in Shanghai, China. Int J Epidemiol. 40, 1510–1518 (2011). 22158661 10.1093/ije/dyr134 PMC3235022

[b15] Jacobsen B. K., Knutsen S. F., Oda K. & Fraser G. E. Parity and Total, Ischemic Heart Disease and Stroke Mortality. The Adventist Health Study, 1976–1988. Eur J Epidemiol. 26, 711–718 (2011). 21701914 10.1007/s10654-011-9598-x PMC3186890

[b16] Koski-Rahikkala H., Pouta A., Pietilainen K. & Hartikainen A. L. Does Parity Affect Mortality Among Parous Women? J Epidemiol Community Health. 60, 968–973 (2006). 17053286 10.1136/jech.2005.044735 PMC2465489

[b17] Steenland K., Lally C. & Thun M. Parity and Coronary Heart Disease Among Women in the American Cancer Society CPS II Population. Epidemiology. 7, 641–643 (1996). 8899393 10.1097/00001648-199611000-00014

[b18] Cooper G. S. *et al.* Menstrual and Reproductive Risk Factors for Ischemic Heart Disease. Epidemiology. 10, 255–259 (1999). 10230834

[b19] Jaffe D. H., Eisenbach Z. & Manor O. The Effect of Parity On Cause-Specific Mortality Among Married Men and Women. Matern Child Health J. 15, 376–385 (2011). 20300814 10.1007/s10995-010-0591-x

[b20] Jaffe D. H., Neumark Y. D., Eisenbach Z. & Manor O. Parity-Related Mortality: Shape of Association Among Middle-Aged and Elderly Men and Women. Eur J Epidemiol. 24, 9–16 (2009). 19145406 10.1007/s10654-008-9310-y

[b21] Zhang X. *et al.* Pregnancy, Childrearing, and Risk of Stroke in Chinese Women. Stroke. 40, 2680–2684 (2009). 19461027 10.1161/STROKEAHA.109.547554 PMC2737806

[b22] Saarelainen H. *et al.* Flow Mediated Vasodilation and Circulating Concentrations of High Sensitive C-reactive Protein, Interleukin–6 and Tumor Necrosis Factor-Alpha in Normal pregnancy–The Cardiovascular Risk in Young Finns Study. Clin Physiol Funct Imaging. 29, 347–352 (2009). 19489963 10.1111/j.1475-097X.2009.00877.x

[b23] Wells G. A. *et al.* The Newcastle-Ottawa Scale (NOS) for Assessing the Quality of Nonrandomised Studies in Meta-Analyses. (2015) http://www.ohri.ca/programs/clinical_epidemiology/oxford.asp. (Date of access: 1/April/2015).

[b24] Stroup D. F. *et al.* Meta-Analysis of Observational Studies in Epidemiology: A Proposal for Reporting. Meta-Analysis of Observational Studies in Epidemiology (MOOSE) Group. JAMA. 283, 2008–2012 (2000). 10789670 10.1001/jama.283.15.2008

[b25] Higgins J. P. & Thompson S. G. Quantifying Heterogeneity in a Meta-Analysis. Stat Med. 21, 1539–1558 (2002). 12111919 10.1002/sim.1186

[b26] DerSimonian R. & Laird N. Meta-Analysis in Clinical Trials. Control Clin Trials. 7, 177–188 (1986). 3802833 10.1016/0197-2456(86)90046-2

[b27] Hamling J., Lee P., Weitkunat R. & Ambuhl M. Facilitating Meta-Analyses by Deriving Relative Effect and Precision Estimates for Alternative Comparisons From a Set of Estimates Presented by Exposure Level Or Disease Category. Stat Med. 27, 954–970 (2008). 17676579 10.1002/sim.3013

[b28] Greenland S. & Longnecker M. P. Methods for Trend Estimation From Summarized Dose-Response Data, with Applications to Meta-Analysis. Am J Epidemiol. 135, 1301–1309 (1992). 1626547 10.1093/oxfordjournals.aje.a116237

[b29] Orsini N., Li R., Wolk A., Khudyakov P. & Spiegelman D. Meta-Analysis for Linear and Nonlinear Dose-Response Relations: Examples, an Evaluation of Approximations, and Software. Am J Epidemiol. 175, 66–73 (2012). 22135359 10.1093/aje/kwr265 PMC3244608

[b30] Guan H. B., Wu L., Wu Q. J., Zhu J. & Gong T. Parity and Pancreatic Cancer Risk: A Dose-Response Meta-Analysis of Epidemiologic Studies. PLoS One. 9, e92738 (2014). 24658609 10.1371/journal.pone.0092738 PMC3962437

[b31] Royston P. A Strategy for Modelling the Effect of a Continuous Covariate in Medicine and Epidemiology. Stat Med. 19, 1831–1847 (2000). 10867674 10.1002/1097-0258(20000730)19:14<1831::aid-sim502>3.0.co;2-1

[b32] Bagnardi V., Zambon A., Quatto P. & Corrao G. Flexible Meta-Regression Functions for Modeling Aggregate Dose-Response Data, with an Application to Alcohol and Mortality. Am J Epidemiol. 159, 1077–1086 (2004). 15155292 10.1093/aje/kwh142

[b33] Egger M., Davey S. G., Schneider M. & Minder C. Bias in Meta-Analysis Detected by a Simple, Graphical Test. BMJ. 315, 629–634 (1997). 9310563 10.1136/bmj.315.7109.629 PMC2127453

[b34] Begg C. B. & Mazumdar M. Operating Characteristics of a Rank Correlation Test for Publication Bias. Biometrics. 50, 1088–1101 (1994). 7786990

