# Supplementary material for: Parity and Cardiovascular Disease Mortality: a Dose-Response Meta-Analysis of Cohort Studies
Source: Sci Rep. 2015 Aug 24;5:13411. doi: 10.1038/srep13411 (PMC4547137; doi:10.1038/srep13411)

Parity and Cardiovascular Disease Mortality: a Dose-Response Meta-Analysis of Cohort Studies

Haichen Lv, Hongyi Wu, Jiasheng Yin, Juying Qian & Junbo Ge

Supplementary Table S1. Methodological quality of cohort studies included in the meta-analysis*

First author, publication year	Representativeness of the exposed cohort	Selection of the unexposed cohort	Ascertainment of exposure	Outcome of interest not present at start of study	Control for important factor or additional factor†	Assessment of outcome	Follow-up long enough for outcomes to occur‡	Adequacy of follow-up of cohorts§
Dior[10], 2013	☆	☆	☆	☆	☆	☆	☆	☆
Simons[12], 2012	☆	☆	☆	☆	☆	☆	☆	—
Jacobs[11], 2012	☆	☆	☆	☆	☆	☆	☆	☆
Jaffe[19], 2011	☆	☆	☆	☆	—	☆	☆	—
Jacobsen[15], 2011	—	☆	☆	☆	—	☆	☆	☆
Gallagher[14], 2011	—	☆	☆	☆	—	☆	☆	☆
Chang[13], 2011	—	☆	☆	☆	☆☆	☆	☆	—
Koski-Rahikkala[16], 2006	☆	☆	☆	☆	☆☆	☆	☆	☆
Cooper[18], 1999	☆	☆	☆	☆	—	☆	—	☆
Steenland[17], 1996	☆	☆	☆	☆	☆☆	☆	—	☆

* A study could be awarded a maximum of one star for each item except for the item Control for important factor or additional factor. The definition/explanation of each column of the Newcastle-Ottawa Scale is available from (http://www.ohri.ca/programs/clinical_epidemiology/oxford.asp).

† A maximum of 2 stars could be awarded for this item. Studies that controlled for body mass index received one star, whereas studies that controlled for other important confounders such as hypertension, cigarette smoking received an additional star.

‡ A cohort study with a follow-up time >10 y was assigned one star.

§ A cohort study with a follow-up rate >75% was assigned one star.

Supplementary Figure S1: Egger's publications bias plot corresponding to the random-effects meta-analysis of the relationship between parity and cardiovascular disease mortality.

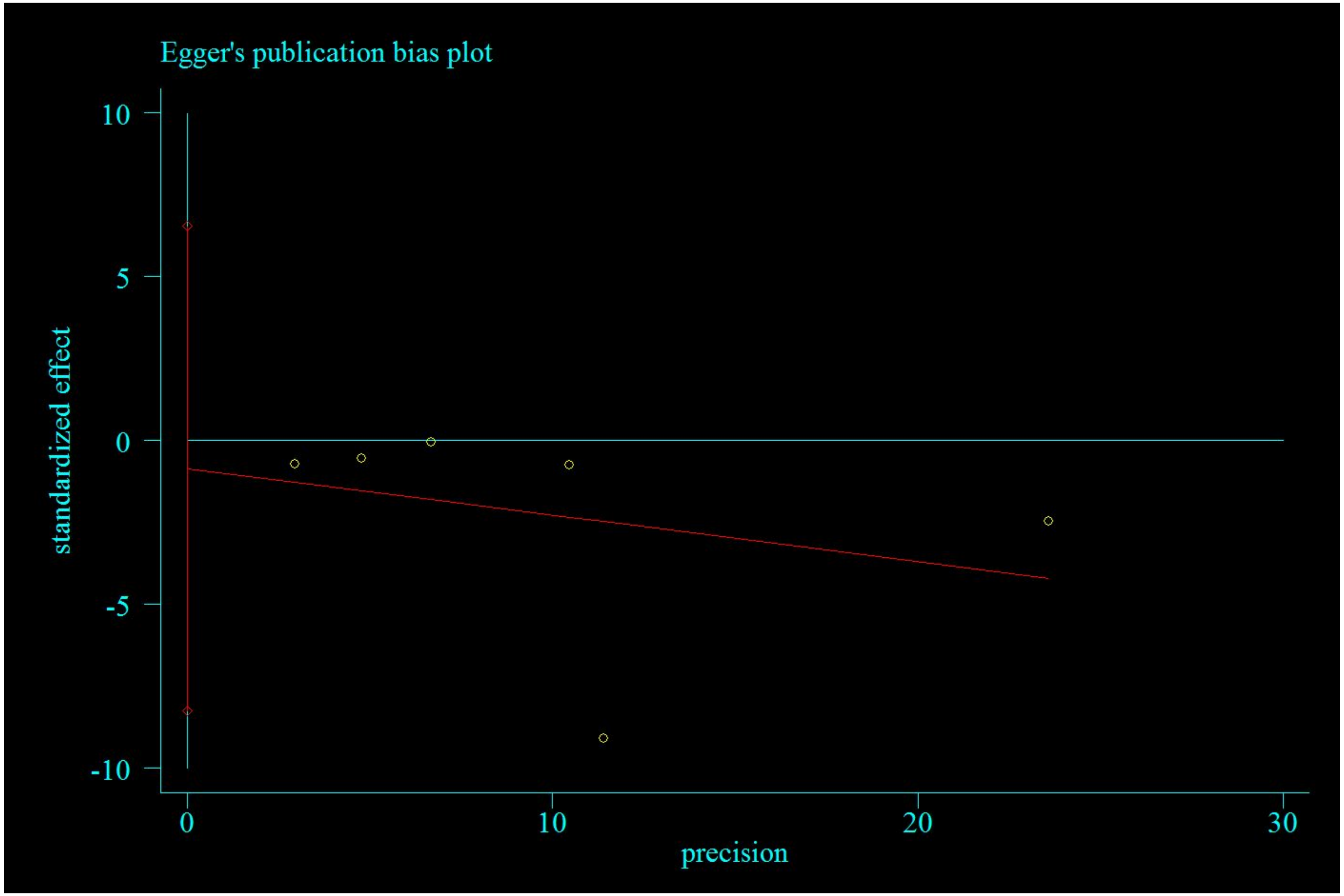

Supplement: Supplementary Information [file srep13411-s1.pdf]
